# The first genetic characterization of *Setaria marshalli* (Nematoda, Spirurida) with reliable DNA barcoding based on a mitochondrial genetic marker

**DOI:** 10.1051/parasite/2022054

**Published:** 2022-11-09

**Authors:** Chihiro Kitajima, Toshihiro Ichijo, Madoka Ichikawa-Seki

**Affiliations:** Cooperative Department of Veterinary Medicine, Faculty of Agriculture, Iwate University Iwate 020-8550 Japan

**Keywords:** *Setaria marshalli*, Filarial nematode, Morphology, DNA barcode, *COI*

## Abstract

*Setaria marshalli* is a mosquito-borne filarial nematode that causes infection in calves younger than two years old. In the present study, nematodes were obtained from a calf in Japan and morphologically identified as *S. marshalli*. Additionally, the partial cytochrome oxidase subunit I (*COI*) region (596 bp) was analyzed for the first time to establish a reliable DNA barcode. Nucleotide sequences of *COI* were identical among the seven worms obtained. The *COI* region can be a useful marker for species discrimination in the case of *S. marshalli* since nucleotide variations observed between the closest congener, *Setaria cervi* (51/596 bp), were sufficient to allow species discrimination. However, the phylogenetic relationship of *S. marshalli* with its congeners was unclear in a maximum likelihood tree. We found that the partial *COI* sequence of *S. marshalli* analyzed in the present study matched a relevant section of the complete mitochondrial genome of *S. labiatopapillosa* that was deposited in the International Nucleotide Sequence Database. This finding suggests that *S. marshalli* was misdiagnosed as *S. labiatopapillosa* in a previous study. It is crucial to conduct accurate morphological analyses to obtain reliable molecular information regarding *Setaria* nematodes.

## Abstract (fr)

*Setaria marshalli* est une filaire transmise par les moustiques qui provoque une infection chez les veaux de moins de deux ans. Dans la présente étude, les nématodes ont été obtenus à partir d’un veau au Japon et identifiés morphologiquement comme *S. marshalli*. De plus, la région partielle de la sous-unité I (COI) de la cytochrome oxydase (596 pb) a été analysée pour la première fois afin d’établir un code-barres ADN fiable. Les séquences nucléotidiques de COI étaient identiques parmi les sept vers obtenus. La région COI peut être un marqueur utile pour la discrimination des espèces dans le cas de *S. marshalli* puisque les variations de nucléotides observées avec le congénère le plus proche, *Setaria cervi* (51/596 pb) étaient suffisantes pour permettre la discrimination des espèces. Cependant, la relation phylogénétique de *S. marshalli* avec ses congénères n’était pas claire dans un arbre à maximum de vraisemblance. Nous avons constaté que la séquence COI partielle de *S. marshalli* analysée dans la présente étude correspondait à une section pertinente du génome mitochondrial complet de *S. labiatopapillosa* qui a été déposée dans la base de données internationale de séquences de nucléotides. Cette découverte suggère que *S. marshalli* a été diagnostiqué à tort comme *S. labiatopapillosa* dans une étude précédente. Il est crucial de mener des analyses morphologiques précises pour obtenir des informations moléculaires fiables concernant les nématodes du genre *Setaria*.

## Introduction

Worms of the genus *Setaria* are mosquito-borne filarial nematodes found in the abdominal cavity of cattle and other ungulates. *Setaria digitata*, *S. labiatopapillosa*, and *S. marshalli* infect cattle, in which adult worms are generally nonpathogenic. However, infective larvae transmitted by mosquitoes to non-natural hosts, including goats, sheep, and horses, fail to migrate to the site of infection and cause cerebrospinal seteriasis, a serious and often fatal neuropathological disorder [6, 8].

*Setaria marshalli* may show transplacental infection in cattle and is typically detected in calves younger than two years old [2, 15]. Adult worms inhabiting the abdominal cavity of calves can cause fibrinous inflammation in the host diaphragm, peritoneum, and omentum [2]. The unique life cycle of the species is highlighted by the congenital intrauterine infection of cow fetuses [13]. However, studies on the pathogenicity of *Setaria* parasites are limited due to the lack of clear symptoms being present in definitive hosts [9], and basic information regarding the parasite is required, including its prevalence in calves [16].

*Setaria digitata*, *S. labiatopapillosa*, and *S. marshalli* can be differentiated based on morphological features [2, 4, 7, 8, 10, 11, 13, 14]. However, misidentification may occur without specialized knowledge and parasitological techniques [13]. Moreover, no DNA sequence for *S. marshalli* is available in the International Nucleotide Sequence Database (INSD). Therefore, it is important to establish a precise DNA barcode for this species to enable further epidemiological studies using molecular tools. Furthermore, such genetic information is valuable to clarify the phylogenetic relationships between *S*. *marshalli* and its congeners.

Here, filarial nematodes obtained from a calf in Japan were identified as *S*. *marshalli* based on morphological features. Moreover, the cytochrome oxidase subunit I (*COI*) of the mitochondrial DNA (mtDNA) was sequenced for the first time to establish a DNA barcode that can be used for species identification as well as to evaluate the phylogenetic relationships between *Setaria* nematodes.

## Materials and methods

This study was conducted in strict accordance with the recommendations of the Guide for the Care and Use of Laboratory Animals of Iwate University. The study protocol was approved by the Committee on the Ethics of Animal Experiments of Iwate University (permission No. A201944).

Seven filarial nematodes (one male and six females) were collected from the abdominal cavity of a 2-month-old male calf (Holstein, born in Morioka, Iwate, Japan) necropsied in January 2021. The worms were killed and uncoiled in hot water to preserve their morphology and stored in 70% ethanol. Morphological identification was performed using one male and two females. After measuring the total length of each worm, a small piece of the midbody was cut for DNA extraction. The remaining parts were cleared in lactophenol (40 mL glycerin, 20 mL lactic acid, 20 mL phenol, and 20 mL distilled water). Morphological structures at the anterior and posterior ends of the worms were observed using a light microscope and measured using ImageJ software [12]. The results of morphological analyses were compared with those of previous studies [2, 8, 14].

Molecular analysis was performed for all seven worms. DNA was extracted from samples using a High Pure polymerase chain reaction (PCR) Template Preparation Kit (Roche, Mannheim, Germany), following the manufacturer’s instructions, and stored at −20 °C prior to analysis. A partial *COI* region of the mitochondrial genome was amplified by PCR reactions using the following primers: COIintF (5′–TGATTGGTGGTTTTGGTAA–3′) and COIintR (5′–ATAAGTACGAGTATCAATATC–3′) [1]. PCR reactions were performed in 25 μL consisting of 2 μL of DNA template, 0.75 μL of each primer (10 μM), 12.5 μL of 2 × Gflex buffer, and 0.625 U of Tks Gflex DNA polymerase (Takara Bio Inc, Shiga, Japan). PCR cycling conditions were as follows: denaturation at 94 °C for 60 s; 40 cycles of 98 °C for 10 s, 52 °C for 15 s, and 68 °C for 30 s; followed by preservation at 10 °C. The PCR products were purified using a NucleoSpin Gel and PCR Clean-up Kit (Takara Bio Inc, Shiga, Japan) and directly sequenced in both directions using a Big Dye Terminator v3.1 Cycle Sequencing Kit (Applied Biosystems, Foster City, CA, USA) on a 3500 Genetic Analyzer (Applied Biosystems). The representative sequence was deposited at the INSD under the accession number LC719467.

The obtained *COI* sequences were aligned with those of *Setaria* worms found in the INSD using GENETYX v15 (Genetyx, Tokyo, Japan). A maximum likelihood (ML) phylogenetic tree was constructed using MEGA-X software [5], with the *COI* sequence of *Wuchereria bancrofti* (AJ271612) used as an outgroup. MEGA-X determined the Tamura–Nei model (+ G) to be the best fit for our dataset and we used it for ML phylogenetic tree construction. Nonparametric bootstrap replication (1000 replicates) was applied to assess nodal support of the phylogeny.

## Results

**Figure 1:**
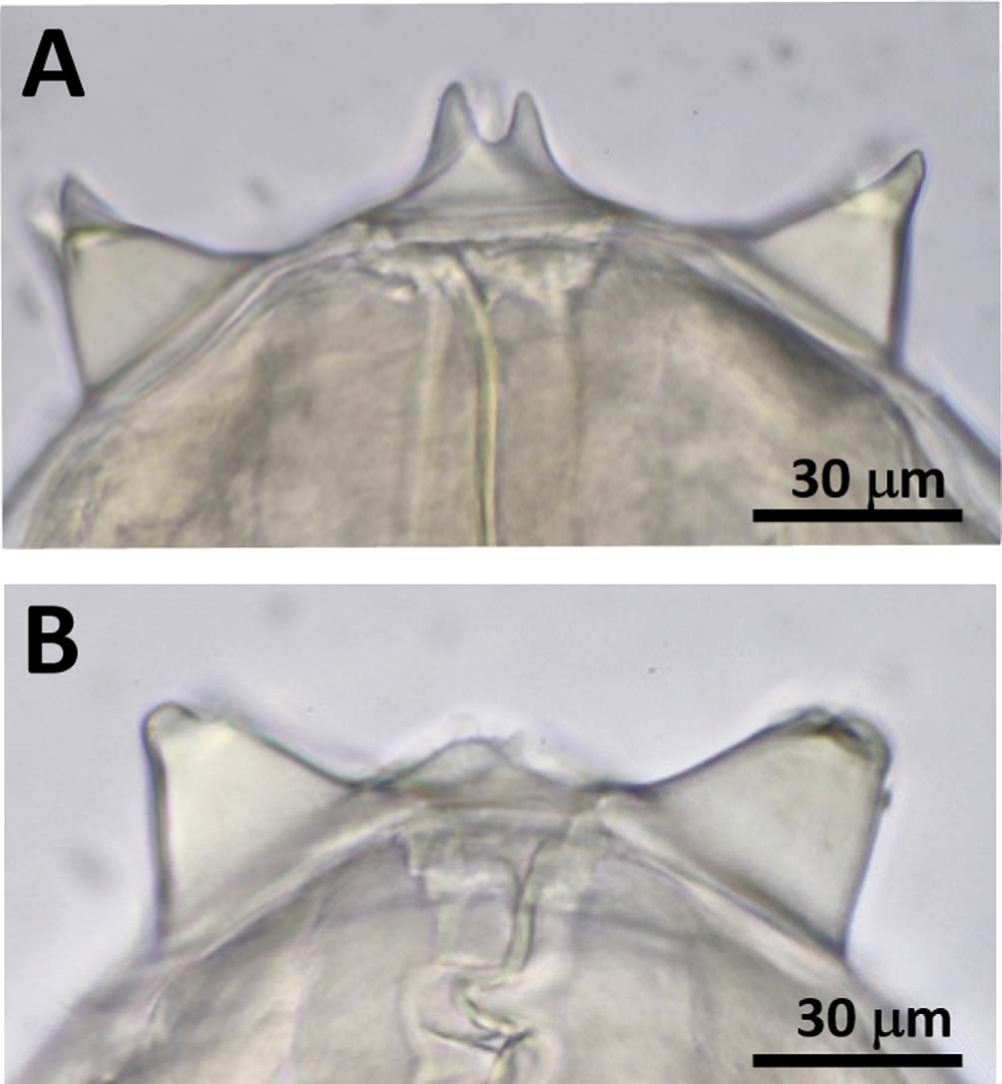
(A) Lateral lips of a female and (B) a male nematode.

**Figure 2:**
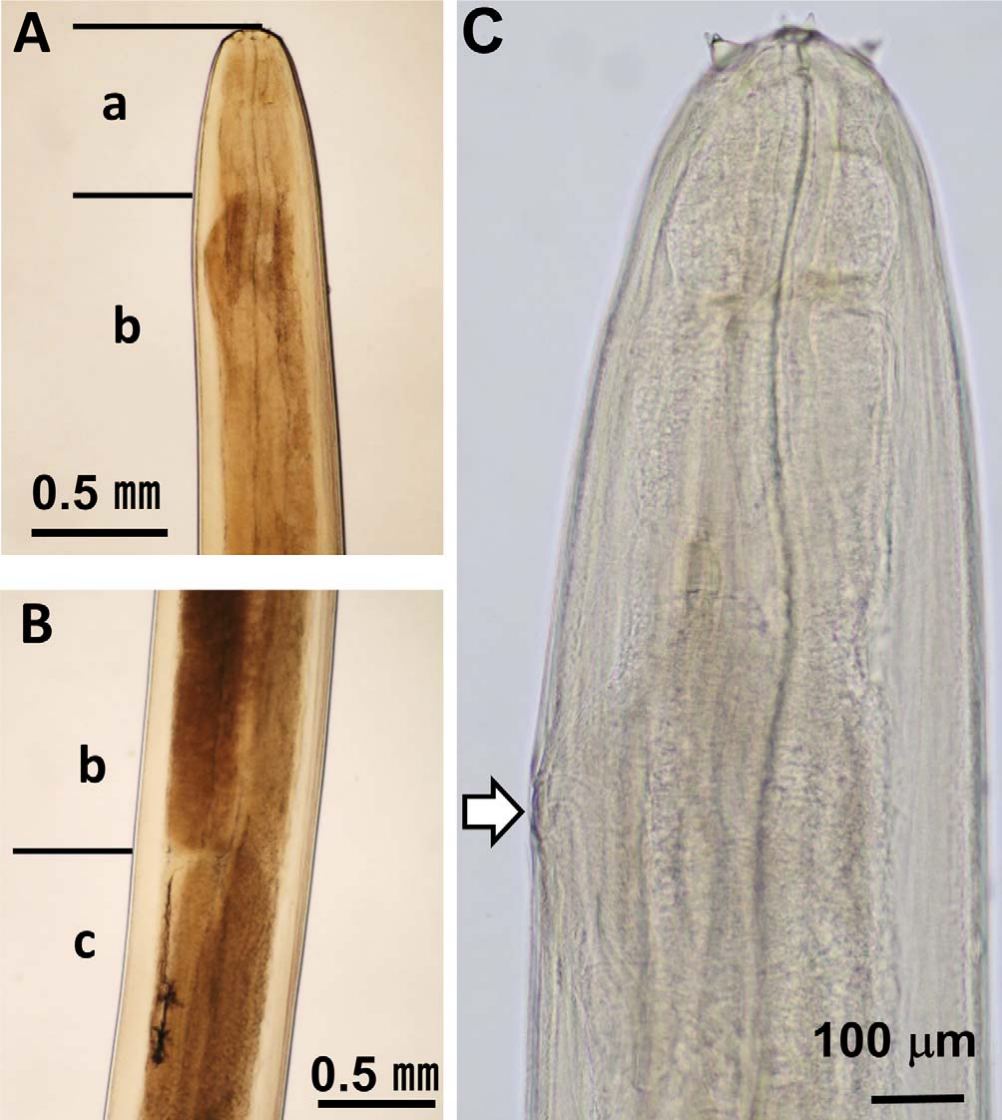
Esophagus of a nematode. (A) The border between (a) the muscular and (b) the glandular part of the esophagus. (B) The border between (b) the posterior end of the glandular part of the esophagus and (c) the intestinal canal. (C) The anterior esophagus of a female. Arrow indicates the vulval opening.

**Figure 3:**
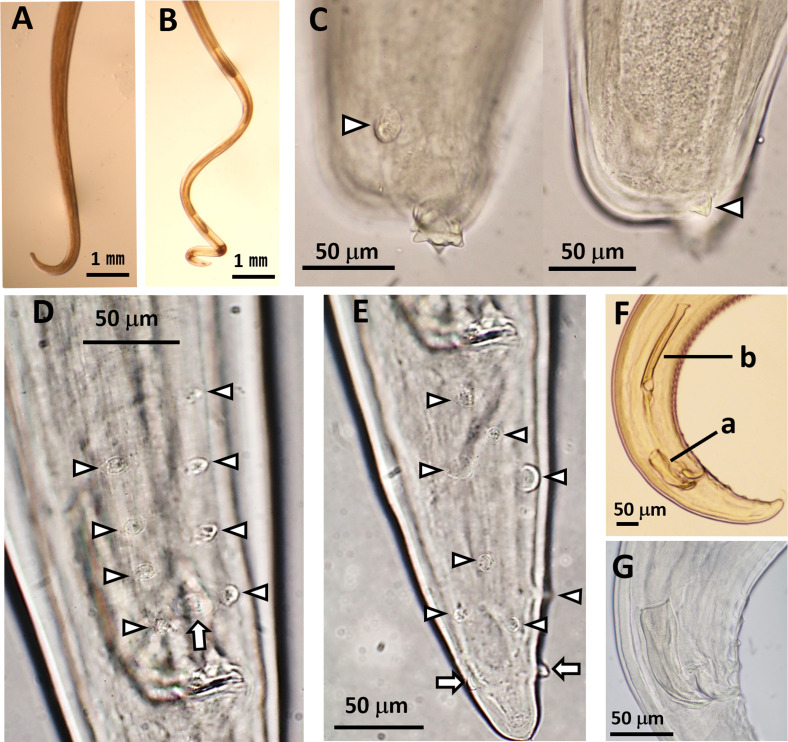
General view of the posterior parts of the nematodes. (A) A female and (B) male nematode. (C) Arrowheads indicate a pair of lateral caudal appendages of a female. (D) The cloacal anterior side of a male with four pairs of precloacal papillae (arrowheads) and one median papilla anterior to the cloaca (arrow). (E) The cloacal posterior side of a male with four pairs of post-cloacal papillae (arrowheads) and a pair of lateral caudal appendages (arrows). (F) (a) The right and (b) left spicules of the posterior end of a male. (G) The right spicule of a male.

The anterior end of each worm was enclosed by a peri-buccal crown with cuticular projections (denticles). The females and male had bifid (Fig. 1A) and simple (Fig. 1B) lateral lips, respectively. The esophagus comprised anterior muscular and posterior glandular parts (Figs. 2A and 2B). The vulva of females was at the esophageal region (Fig. 2C). The posterior end was loosely coiled in females (Fig. 3A) and tightly coiled in the male (Fig. 3B). The caudal end of females possessed a terminal knob with several spines and a pair of lateral caudal appendages (Fig. 3C). In contrast, the caudal end of the male displayed four pairs of precloacal papillae, a single median papilla anterior to the cloaca, four pairs of post-cloacal papillae, and lateral caudal appendages on both sides (Figs. 3D and 3E). Moreover, there was slight asymmetry in the arrangement of both pre- and post-cloacal papillae in the male (Figs. 3D and 3E). The right and left spicules of the male differed in length and shape, with the right one being short and stout and the left one being long and slender with a pointed tip (Figs. 3F and 3G). Based on the morphological features described above, the filarial nematodes obtained in this study were identified as *S. marshalli*. The morphometry of the parasites along with those of *S. marshalli* from previous studies are shown in Table 1, while that of *S. digitata* and *S. labiatopapillosa* are presented in Table S1. *Setaria marshalli* can be distinguished from these two congeneric species by the shape of the lateral lips (bifid) in females and by the numbers and positions of papillae at the caudal ends of males.

**Table 1:** Morphological characteristics of *Setaria marshalli.*

			*S. marshalli*	
	Our samples		Taniguchi 1953/Nakano *et al*. 2007/Fujii *et al.* 1995	
	Female (*n* = 2)	Male (*n* = 1)	Female	Male
Length (cm)	9.1–9.5*	5.3	11.7/7.2–11.0/4.2–12.1	5.8/4.8–5.8/3.9–5.5
Width (mm)	0.75–0.77	0.46	0.63/0.62–0.78/0.52–0.75	0.48/0.44–0.46/0.44–0.53
Distance between the dorsal and ventral spines (μm)	142–162	78	194–216/–/111–161	–/–/94–104
Lateral lip	Bifid	Non-bifid	Bifid/–/resembled teeth	Non-bifid/–/triangular-shaped
Length of the esophagus (mm)	10.65–10.74	8.42	–	–
Muscular part (mm)	0.67–0.70	0.92	–	–
Glandular part (mm)	9.98–10.05	7.50	–	–
Anterior end to vulva (μm)	0.75–0.84	–	0.8/–/–	–
Length of lateral caudal appendages (μm)	10.31–10.59	–	5.4–32.4/–/–	–
Posterior end to lateral caudal appendages (μm)	74.58–77.68	–	27–81/–/–	–
Posterior end	Terminal knob with several spines	• Four pairs of precloacal papillae	Terminal knob with several spines/roughly furcated/–	• Four pairs of precloacal papillae
		• A single median papilla anterior to the cloaca		• A single median papilla anterior to the cloaca
		• Four pairs of post cloacal papillae		• Four pairs of post cloacal papillae
		• Lateral caudal appendages on both sides near the caudal end		• Lateral caudal appendages on both sides near the caudal end
Length of left sexual spicules (μm)	–	0.24	–	0.23/–/–
Length of right sexual spicules (μm)	–	0.13	–	0.07/–/–

* *n* = 6.

**Figure 4:**
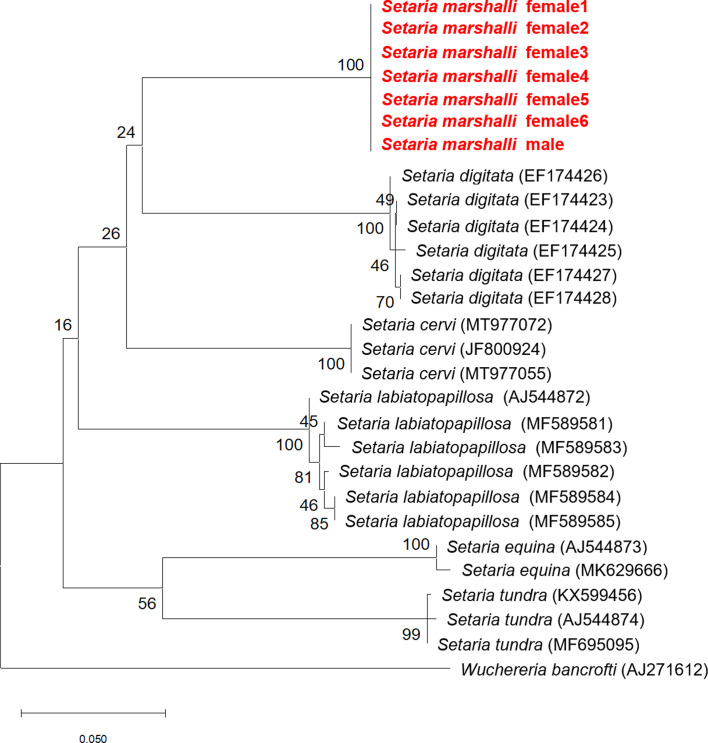
Maximum likelihood (ML) phylogenetic tree based on nucleotide sequences of mitochondrial DNA of the cytochrome oxidase subunit I (*COI*) region. *Setaria marshalli* is shown in bold red. All sequences of the species obtained in this study were identical and deposited at the INSD under accession No. LC719467.

Partial sequences (596 bp) of the *COI* of the seven nematodes were identical, and they were most similar to that of *S. cervi* (MK360913) (545/596 bp, identity: 91%). The single nucleotide polymorphisms (SNPs) detected among the congeners are shown in Figure S1. However, nodal supports within the ML phylogenetic tree (Fig. 4) were low.

## Discussion

The measurements and morphological characteristics of the worms (Table 1) were consistent with those described for *S. marshalli* in previous studies [8, 14], except for the measurement of the right spicule (Table 1). However, the study referred to [14] provided a mean value for this structure, whereas the right spicule was measured from a single male specimen in the present study. Therefore, such a mismatch in results is a product of individual differences among worms.

The mtDNA sequence of the *COI* region is a useful species differentiation marker for *S. marshalli*; it was identical among all the samples and displayed adequate differences (545/596 bp) to that of the most closely related species, *S. cervi* (Fig. S1). This DNA barcoding of *S. marshalli* will play an important role in further epidemiological studies.

The phylogenetic relationships between *S. marshalli* and other *Setaria* species were analyzed for the first time. However, their relationships remain unclear because nodal supports within the *Setaria* clade were too low (Fig. 4). Further analyses that use longer sequences are required to reveal the relationships among *Setaria* nematodes.

In fact, the partial sequence of the *COI* region used in this study completely matched that section of the complete mitochondrial genome of *S. labiatopapillosa* (MH937750), as found in the INSD [3]. However, as there were no morphological species identification or morphometric measurements of organs in the previous study [3], it is likely that *S. marshalli* was misidentified as *S. labiatopapillosa*. Therefore, DNA analysis should not be performed without accompanying accurate morphological identification. It is extremely important to conduct morphological and molecular analyses simultaneously to obtain accurate molecular information on worms.

In conclusion, we performed molecular characterization for *S. marshalli* for the first time, and an accurate DNA barcode was successfully obtained for the species. The results of the present study will contribute to epidemiological surveys of *Setaria* nematodes in the future.

## Supplementary files

The supplementary material of this article is available at https://www.parasite-journal.org/10.1051/parasite/2022054/olm.**Figure S1:**
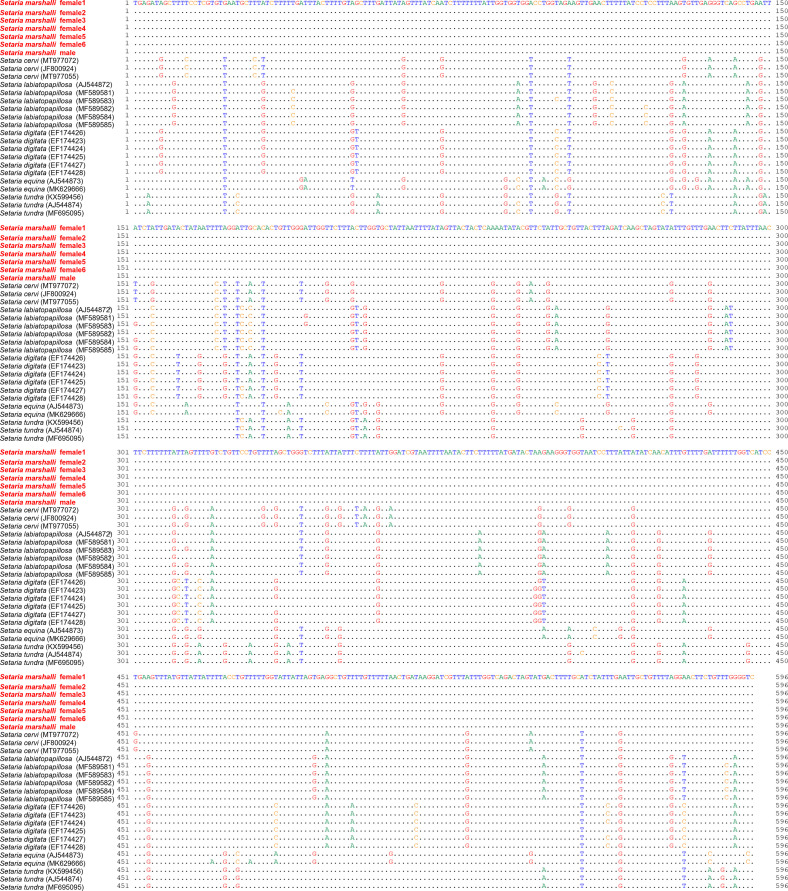
Alignment of mitochondrial DNA of the cytochrome oxidase subunit I (*COI*) region. The nucleotide sequences used in the maximum likelihood (ML) tree were included. A dot in the alignment indicates that the sequence was identical to that of *S. marshalli*.*Table S1*: Morphological characteristics and measurements of the *Setaria* nematodes.
